# Complete genome sequences of *Thermus* strains isolated from Kinosaki Hot Spring in Japan

**DOI:** 10.1128/mra.00315-26

**Published:** 2026-07-29

**Authors:** Haruki Omichi, Yuki Takafuji, Kentaro Miyazaki, Hiroya Tomita, Kohsuke Honda

**Affiliations:** 1 Graduate School of Engineering, The University of Osaka 13013, Suita, Osaka, Japan; 2 International Center for Biotechnology, The University of Osaka 13013, Suita, Osaka, Japan; 3 Industrial Biotechnology Initiative Division, Institute for Open and Transdisciplinary Research Initiatives (OTRI), The University of Osaka 13013, Suita, Osaka, Japan; University of Wisconsin-Madison, Madison, Wisconsin, USA

**Keywords:** *Thermus thermophilus*, *Thermus brevis*, *Thermus brockianus*, complete genome sequences

## Abstract

Here, we report the complete genome sequences of two *Thermus thermophilus*, one *Thermus brockianus*, and two *Thermus brevis* strains isolated from Kinosaki Hot Spring, Japan, generated using a hybrid assembly approach combining Oxford Nanopore long-read and Illumina short-read data.

## ANNOUNCEMENT

Over half a century ago, thermophilic bacteria thriving in near-boiling water were successively isolated: *Thermus aquaticus* from Yellowstone National Park in the United States (1) and *Thermus thermophilus* from hot springs in Japan (2, 3). Since then, *Thermus* bacteria have attracted much attention as a source of thermostable enzymes (4–9). *Thermus* is also characterized by its natural competency (10), leading to the early development of genetic manipulation systems (10, 11). In particular, *T. thermophilus*, which grows optimally at around 75°C, has been studied as a model thermophilic organism (12). To date, numerous *Thermus* strains have been isolated from geothermal environments worldwide (13–23). In recent years, we have isolated numerous *Thermus* strains from hot springs across Japan, discovering plasmids with new replication origins (21) and developing a new series of shuttle vector (24). Here, we report the complete genome sequences of five *Thermus* strains isolated from Kinosaki Hot Spring, Japan (35.62587005°N, 134.80416244°E).

A hot spring water sample (200 µL) was spread onto TT (8 g L^−1^ tryptone, 4 g L^−1^ yeast extract, and 2 g L^−1^ NaCl) or R2A (25) medium plates solidified with agar (1.6 g L^−1^) and incubated at 65°C overnight. As a result, dozens of well-isolated colonies were obtained, and their 16S rRNA genes were amplified by colony PCR (26) and sequenced. All isolates belonged to the genus *Thermus*, from which five strains, designated KS1, KS13, KS21, RAM1-1, and RAM3-1, were selected for subsequent whole-genome analysis.

To prepare genomic DNA, cells were grown in 5 mL of TT medium at 65°C for 48 h with shaking at 180 rpm. Genomic DNA was purified using a blood and cell culture DNA minikit (Qiagen), following the manufacturer’s protocol. For long-read sequencing, 1 µg of unsheared genomic DNA was treated with a short-read eliminator kit (Circulomics) to remove <10 kbp fragments and used for library preparation with the Native Barcoding Kit (SQK-NBD114-24; Oxford Nanopore Technologies) following the manufacturer’s protocol and sequenced on a MinION platform with an FLO-MIN114 flow cell. Base calling was performed using Dorado v. 1.0.0 in super-accuracy (SUP) mode. The raw sequencing data were filtered (Q < 10; length <1,000 bases) using NanoFilt v.2.7.1 (27). For short-read sequencing, paired-end libraries were prepared using an Illumina PCR-free library preparation kit following the manufacturer’s protocol and sequenced on a NovaSeq X Plus platform (2 × 151 bp). Adapter trimming was performed using cutadapt v. 1.9.2 (28), followed by hybrid assembly with Flye v. 2.9.1-b1780 (29) and polishing with Pilon v. 1.23 (30); the circularity was confirmed using Unicycler v.0.4.8 (31). Completeness was assessed using CheckM v.1.2.2 (32), implemented in DFAST v.1.6.0 (33). Default parameters were used, unless otherwise specified.

Automatic annotation was conducted using DFAST v.1.6.0 (33). Genome statistics are summarized in Table 1. A genome-based taxonomic analysis on the TYGS server (34) revealed that KS1 and KS13 belong to *T. thermophilus* (the average nucleotide identity [ANI] calculated using a JSpecies analysis [35] was 97.39% with HB8^T^), KS21 belongs to *T. brockianus* (the ANI was 98.37% with GE-1^T^), and RAM1-1 and RAM3-1 belong to *T. brevis* (the ANI was 97.52% with SYSU G05001^T^). As the complete genome sequence of *T. brevis* does not exist, the genome sequences of RAM1-1 and RAM3-1 are the first complete genomes of this species. Among the five isolates, *T. thermophilus* (KS1 and KS21) and *T. brockianus* (KS13) contained a large circular DNA in each strain. These large circular DNAs (pTthKS1b, pTbroKS13b, and pTthKS21b) were considered megaplasmids based on the homology to known megaplasmids identified in *Thermus* species. Notably, however, *T. brevis* strains (RAM1-1 and RAM3-1) lacked such a large circular DNA, and the genes involved appear to be integrated into the chromosome. In pTbroKS13c, a putative replication protein (TbroKS13_26220) and a putative ParA protein (TbroKS13_26320) were identified, whose homologs are widely distributed in *Thermus* plasmids. In pTbroKS13d, a putative replication protein (TbroKS13_26850) was identified, whose homologs are also widely distributed in *Thermus* plasmids. Therefore, these relatively small circular DNAs may be plasmids.

**TABLE 1:** Sequencing metrics for the five *Thermus* strains in this study

Strain	BioSample accession no.	NovaSeq X Plus (short read)				MinION (long read)					Complete genome			
		No. of paired-end reads	Total length (Mb)	Accession no.	Avg read depth (×)	No. of reads	N50 (bases)	Total length (Mb)	Accession no.	Avg read depth (×)	Length (bp)	G + C (%)	No. of CDS	Accession no.
*Thermus thermophilus* KS1	SAMD01824206	8,988,609	18.0	DRR911462		65,611	38,773	300.7	DRR911467					
Chromosome					1,028.3					140.9	1,968,983	69.1	2,119	AP046144
pTthKS1b					1,181.3					173.3	243,718	68.5	272	AP046145
*Thermus brockianus* KS13	SAMD01824207	8,094,432	16.2	DRR911463		76,984	49,626	388.6	DRR911468					
Chromosome					802.1					142.7	2,139,201	67.0	2,292	AP046146
pTbroKS13b					1,111.8					148.1	328,879	70.2	8	AP046147
pTbroKS13c					2,537.9					300.4	21,020	68.4	62	AP046148
pTbroKS13d					3,787.0					318.6	9,055	66.0	328	AP046149
*Thermus thermophilus* KS21	SAMD01824208	8,034,106	16.1	DRR911464		48,744	34,508	214.4	DRR911469					
Chromosome					940.2					98.0	1,970,092	69.1	2,119	AP046150
pTthKS21b					989.0					82.7	243,718	68.5	273	AP046151
*Thermus brevis* RAM1-1	SAMD01824209	7,259,412	14.5	DRR911465		73,053	54,450	291.6	DRR911470					
Chromosome					821.0					128.3	2,281,347	65.0	2,365	AP046152
*Thermus brevis* RAM3-1	SAMD01824210	7,952,199	15.9	DRR911466		83,781	50,775	368.3	DRR911471					
Chromosome					865.9					160.5	2,282,668	65.0	2,372	AP046153

## Data Availability

All *Thermus* strains reported in this paper are associated with BioProject PRJDB40431. The genome sequences and raw sequencing data are available under the accession numbers listed in Table 1.
